# Identification of a Novel Densovirus in Aphid, and Uncovering the Possible Antiviral Process During Its Infection

**DOI:** 10.3389/fimmu.2022.905628

**Published:** 2022-06-09

**Authors:** Tong Li, Haichao Li, Yuqing Wu, Shaojian Li, Guohui Yuan, Pengjun Xu

**Affiliations:** ^1^ Institute of Plant Protection, Henan Key Laboratory of Crop Pest Control, Key Laboratory of Integrated Pest Management on Crops in Southern Region of North China, Henan Academy of Agricultural Sciences, Zhengzhou, China; ^2^ College of Plant Protection, Henan Agricultural University, Zhengzhou, China; ^3^ Key Laboratory of Insect Developmental and Evolutionary Biology, Center for Excellence in Molecular Plant Sciences, Institute of Plant Physiology and Ecology, Chinese Academy of Sciences, Shanghai, China; ^4^ Tobacco Research Institute, Chinese Academy of Agricultural Sciences, Qingdao, China

**Keywords:** densoviruses, immune, *Sitobion miscanthi*, aphid, antiviral process, infection

## Abstract

Densoviruses (DVs) are single-stranded DNA viruses and exclusively happen in invertebrates. Most of DVs reported in insects are pathogenic to their native hosts, however, no pathogenic effect of them has been examined in vertebrates. Hence, DVs are the potential agents used in pest managements. Aphids are the primary vectors of plant viruses. In this study, we identified a novel DV in Chinese *Sitobion miscanthi* population, provisionally named “Sitobion miscanthi densovirus” (SmDV). Taxonomically, SmDV belongs to genus *Hemiambidensovirus*. In *S. miscanthi*, SmDV is hosted in diverse cells and can be horizontally transmitted *via* wheat feeding. Subject to SmDV, aphids activate their intrinsic antiviral autophagy pathway. Grouped with ascorbate and aldarate metabolism, chlorophyll metabolism, p450 related drug metabolism, and retinoid metabolism, aphids form a complex immune network response to the infection of SmDV. Obviously, it works as elder aphids still alive even they contain the highest examined concentration of SmDV. This study provides a foundation for the identifications of novel DVs, and further improves the understanding of the molecular interactions between insects and DVs.

## Introduction

In nature, virus is a major cause of insect mortality (1). During the longtime arms-race, insect innate immune system has been evolved diverse mechanisms resistant to the infection of virus. In *Drosophila*, the intact IMD pathway play key role in response to the infections of Cricket Paralysis Virus (CrPV) and Sindbis virus (SINV) (2). *In vivo*, cellular RNA interference process (RNAi) can effectively inhibit the proliferation of Drosophila C virus (DCV) and Cricket Paralysis Virus (DCV) (3). Cyclic GMP-AMP (cGAMP) synthase (cGAS) is known involved into the antiviral immune response in mammals. Recently, two cGAS-like receptors (cGLRs) are revealed to be broad-spectrum antiviral agents in *Drosophila* (4). To date, using second-generation sequencing technology, plenty of novel insect viruses have been identified (5–7), however, the underlying immune mechanisms subject to their infections in insects are less known.

*Aphids* (Hemiptera: Aphididae) are tiny and soft-bodied insects that significantly affect crop production. It is well known that aphids are the primary carriers of plant viruses (8). In addition to plant viruses, several pathogenic viruses in aphids have been isolated, such as Rhopalosiphum padi virus (RhPV) (9), Acyrthosiphon pisum virus (APV) (10), Brevicoryne brassicae virus (BrBV) (11), and Aphid lethal paralysis virus (ALPV) (12). However, the traditional method in the virus classification would underestimates the virus diversity in aphids. Next generation sequencing technologies (NGS) have facilitated the discovery of invertebrate viruses (5, 13). *Sitobion miscanthi* (Takahashi) is widely distributed in Chinese wheat planting regions (14). The close relationship between *S. miscanthi* and bacterial symbionts have been comprehensively studied (15–17). However, the viral landscape of Chinese *S. miscanthi* is less known. In this study, employed the RNA-seq, we identified a densovirus in *S. miscanthi*, and provisionally named as “Sitobion miscanthi densovirus” (SmDV).

Densoviruses (DVs) are ssDNA viruses and widely distributed in invertebrates (18). Taxonomically, DVs are classified into the subfamily *Densovirinae* of *Parvoviridae*. Previously, five genera have been proposed in *Densovirinae*: *Ambidensovirus, Brevidensovirus*, *Iteradensovirus, Hepandensovirus*, and *Penstyldensovirus* (19, 20). However, in the latest taxonomic proposal of *Densovirinae* provide by ICTV (International Committee on Taxonomy of Viruses, Code assigned: 2019.010D), major taxonomy revisions have been made. *Hepan*-, *Penstyl*- and *Brevidensovirus* are grouped into the new subfamily *Hamaparvovirinae*, and *Ambidensovirus* is divided into several new genera. To date, *Densovirinae* is comprised of 11 genera and 21 officially identified species, including two aphid DVs: Myzus persicae densovirus (MpDV1) (21), and Dysaphis plantaginea densovirus (DplDV1) (22). Both MpDV1 and DplDV1 are pathogens in their native hosts, but DplDV1 can also induce wing development in *Dysaphis plantaginea*.

In this study, the complete SmDV genome was determined. The phylogenetic position, gene expression patterns and population dynamic of it were uncovered. Using *in situ* hybridization method, the cellular infection pattern of SmDV was investigated. Furthermore, in *S. miscanthi*, the gene expression profiles subject to the SmDV infection were uncovered. Combined with gene-concept network analysis, the potential anti-SmDV core genes and their enriched KEGG pathways were filtered. It was mysterious that some metabolism pathways involve in the human antiviral process, would also probably play their roles in insects.

## Materials and Methods

### Aphid Rearing

In our laboratory, about twenty *S. miscanthi* populations collected from different geographical wheat planting regions were reared on wheat seedlings at 20°C with a light:dark cycle of 16:8 hr.

### Aphid RNA-Seq and Densovirus-Like Fragments Extraction

In this study, bulk RNA-seq method was performed in aphids. Five adult aphids from each *S. miscanthi* geographical population were collected and total RNA was extracted with the TRIzol reagent (Invitrogen, Carlsbad, CA, United States). The mRNA was enriched by removing rRNA using the Ribo-ZeroTM Magnetic Kit (Epicentre, Madison, WI, United States). The sequencing library was prepared by Gene Denovo Biotechnology Co. (Guangzhou, China) and sequenced on an Illumina novaseq 6000 platform with paired-end method. Clean reads were filtered by fastp (version 0.18.0) (23) and transcriptome denovo assembly was carried out by Trinity (24), with default parameters. The possible densovirus-like unigenes were filtered with our previous reported methods (6), but employed the protein sequences of *Parvoviridae* (txid10780) as subject in the local blastx searches. The obtained putative densovirus sequences were further comfirmed with online blastx analyses to Non-redundant protein sequences (nr) database. An e-value threshold of 1 × 10^−5^ was used in these searches.

### SmDV Genome Organization, Phylogenetic Analysis and Detection

Full-length cDNA sequences of the SmDV NS and VP genes were obtained using the 3’ and 5’ rapid amplification of cDNA ends (RACE) system (Life Technologies, Carlsbad, CA, United States). The inverted terminal repeat (ITR) of SmDV were further determined by PacBio SMRT sequencing by BGI (Shenzhen, China). The assembled SmDV genome obtained by high throughput techniques were further verified through amplification and sequencing using specific primers (**Table S1**). The DNAs used in PCR were extracted from a single aphid using an Ezup Column Animal Genomic DNA Purification Kit (Sangon Biotech, Shanghai). The open reading frames of SmDV genome were predicted in NCBI online ORF finder program with standard genetic code. Furthermore, conserved domains within the ORFs were predicted in NCBI conserved domain database with threshold of 1 × 10^−2^. The structure of SmDV genome was visualizated by IBS program (25).

The NS1 and VP protein sequences from *Densovirinae* taxa were retrieved to uncover the phylogenetic position of SmDV (**Table S2**). The sequences were aligned using the MUSCLE program in MEGA 7.0 (26) and then trimmed using trimAl to remove the poorly aligned regions (27). Phylogenetic analysis was performed in IQ-TREE 1.6.6 (28), followed by resampling 1,000 ultrafast bootstraps to assess the support for each node. The substitution models were selected based on the Bayesian information criterion in ModelFinder (29). A tanglegram of the two phylogenetic trees was constructed with Dendroscope (30).

The infection of SmDV in room reared *S. miscanthi* geographical populations was detected with specific primers (listed in **Table S1**). And then the SmDV natural positive and negative *S. miscanthi* isofemale strains were built with a founder aphid collected from the Yuanyang (YY-strain) and Luoyang (LY-stain) wheat fields in China, respectively. The five known bacterial symbionts in *S. miscanthi* (i.e., *Serratia symbiotica, Hamiltonella defensa, Regiella insecticola*, *Wolbachia pipientis* and SMLS) were not detected in the both *S. miscanthi* isofemale strains (15, 16, 31).

### Fluorescence *In Situ* Hybridization

The cellular tropism of SmDV in aphid embryos was identified using *in situ* hybridization. The process of *in situ* hybridization was generally followed the methods reported previously (32). Under a stereoscopic microscope, more than 50 intact aphid embryos aphid embryos were dissected from adults of the YY stain in cold 70% ethanol using an insect pin, and then fixed in Carnoy’s solution (chloroform-ethanolacetic acid [6:3:1]) for 10 hours. The fixed embryos were decolorized overnight in alcoholic 6% H2O2 solution, then pre-hybridized three times in hybridization buffer (20 mM Tris-HCl [pH 8.0], 0.9 M NaCl, 0.01% sodium dodecyl sulfate, 30% formamide) for six hours each time. Embryos were then incubated overnight in hybridization buffer containing 100 pmol/ml of each fluorescent probe and 0.5 mg/ml 49,69-diamino-2-phenylindole (DAPI). Finally, the embryos were washed in a buffer (0.3 M NaCl, 0.03 M sodium citrate, 0.01% sodium dodecyl sulfate) and observed under a laser confocal microscope (LSM 510 META, Carl Zeiss). In the hybridization, a reported fluorescent probe was used to target the primary symbiont *Buchnera aphidicola*16S rRNA (33), and new designed fluorescent probe targeted SmDV NS1 mRNA, SmDV-Alexa Fluor 488 (5’-Alexa Fluor 488-TCGTCGTCGACATAATTGGA-3’) were employed. DAPI was used to counterstain the nuclei of aphid cells. No-probe and RNase digestion control experiments were employed to confirm the specificity of the detection. All manipulations were performed at room temperature.

### Quantification of SmDV Within the Development of Aphid

DNA was extracted from a series of aphids of the YY strain according to days after birth. Titers of SmDV were quantified in terms of the NS1 gene copies. Quantitative PCR was performed in StepOnePlus Real-Time PCR System (ABI, USA) using the SYBR Green I method. Forward primer SmDV-NSqF3 (5’-CCTATCTACCGAAGTATG-3’) and reverse primer SmDV-NSqR3 (5’- GAACCGAATATCATCAAC-3’) amplified a 103 bp fragment. Aphid cell concentration was quantified in terms of *ef1a* gene copies with previous reported primers (17). Quantitative PCR reactions were carried out in a 25 μl volume containing 12.5 μl 2×TransStart Green qPCR SuperMix UDG (TransGen, Beijing), 0.5 μl 50×Passive Reference Dye, 10.5 μl sterile water, 0.5 μl of each primer (10 μmol) and 1 μl DNA. Cycling conditions were 50°C for 2 min (UDG enzyme digestion), 94°C for 10 min, followed by 35 cycles at 94°C for 30 s, 55°C for 30 s, 72°C for 30 s. Finally, a melting curve was constructed. Standard curves were constructed with serial dilution plasmids, which contained 10^8^, 10^7^, 10^6^, 10^5^ and 10^4^ copies/μl of *ns1* and 10^8^, 10^7^, 10^6^, 10^5^ and 10^4^ copies/μl of *ef1a*. Sterile water was used as the template in the NTC (no template control).

### SmDV Horizontal Transmission

In this study, the SmDV was horizontally transmitted into the natural negative *S. miscanthi via* feeding on the wheat leaves after fed by YY-strain aphids. Briefly, five adult aphids of YY-strain were fed on one wheat seedling for 72 hours, and then ten of the newborn nymphs from *S. miscanthi* LY strain were fed on the SmDV infected wheat seedling for 48 hours. Thereafter, the nymphs were independently reared on the normal wheat seedling until adult. SmDV was detected in them to evaluate horizontal transmission rate of SmDV *via* wheat feeding. The artificial SmDV positive *S. miscanthi* population was built with the offspring of the SmDV infected ones, which called as LY-SmDV.

### The Effects of SmDV on Aphid Gene Expressions

The aphids collected from LY and LY-SmDV strains were employed into the RNA-seq to uncover the effects of SmDV on the aphid gene expression profiles. The RNA-seq was performed as above mentioned. The unigene expressions were calculated and normalized to RPKM (Reads Per kb per Million reads). The DESeq2 (34) was employed to filter the differentially expressed genes (DEGs) at a threshold absolute fold change (FC)≥4 and false discovery rate (FDR) < 0.05. The annotations of DEGs were searched in Nr, KEGG (Kyoto Encyclopedia of Genes and Genomes) with BLASTx at an e-value threshold of 1 × 10^-5^. The GO (Gene Ontology) annotations were mapped to the GO terms in the Gene Ontology database (http://www.geneontology.org/) with Blast2GO software (35). Principal component analysis (PCA) of the samples was determined with the gene expression matrix in R software. The GO and KEGG enrichment of the DEGs were performed using the OmicShare tools, a free online platform for data analysis (https://www.omicshare.com/tools). The gene-concept network highlighted the interactions between genes and KEGG pathways was further constructed using the clusterProfiler R package (36). The gene expression patterns of the DEGs clustered into the significantly enriched pathways were further verified by qRT-PCR and visualized in the heat maps constructed by TBtools (37). For qRT-PCR, the cDNA was synthesized with first strand cDNA Synthesis Kit (Toyobo, Shanghai), and then performed with SYBR Green Real-time PCR Master Mix Kit (Toyobo) on a Mastercycler^®^ ep realplex (Eppendorf). The relative abundance of DEGs was normalized to the *S. miscanthi* β-actin gene (38), according to the methods described previously (39, 40). All qRT-PCR assays were carried out in three biological replicates, and further analyzed with one-way analysis of variance (ANOVA) method.

## Results

### SmDV Identification and Detection in Wild Aphid Populations

**Figure 1:**
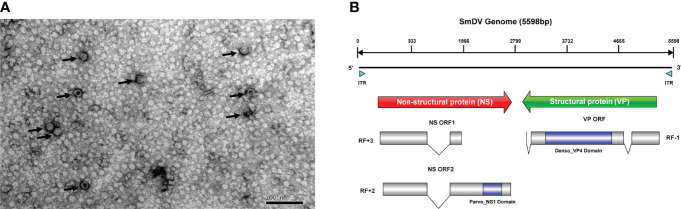
The genome and virion features of SmDV. **(A)** Electron microscopy of purified SmDV particles. **(B)** The genome characteristic of SmDV. ITR, inverted terminal repeat; RF, read frame. Polylines indicate the intron splicing. Arrows point to virus particles.

There were 47,217 unigenes assembled in the *S. miscanthi* bulk transcriptome. Two virus-originated fragments were detected, named as unigene-1 (1788 nucleotides) and unigene-2 (3086 nucleotides), which shared 64.89% and 58.70% similarity to the NS and VP protein sequences of a *Myzus persicae* densovirus (*M. persicae* nicotianae densovirus strain SDMPN, Accession number: KT239104.1). It indicated these fragments probably originated from a densovirus, which tentatively named SmDV. Furthermore, the SmDV particles were enriched and purified with previously reported methods (7), and then observed them under the transmission electron microscope (**Figure 1A**). The result showed that the size of SmDV particle was approximate 20 nm in diameter. The infection pattern of SmDV in wild *S. miscanthi* was evaluated in the geographical aphid populations with specific PCRs. The results indicated that the infection rates of SmDV vary widely among the aphid populations (0-100%) (**Table S3**). In all samples, the infection rate of SmDV was about 54.2% (103/190).

### SmDV Genomic Characterization and Phylogenetic Analysis

The ITR regions of SmDV were not directly amplified, but obtained from the PacBio SMRT sequencing results. The ITR of SmDV was 163 nts in length. The quickmold algorithm was used to predict the secondary structure of SmDV ITR, and hairpin structures were observed in them (**Figure S1**). Including ITR regions, the full length of SmDV was 5598 nucleotides.

SmDV has an ambisense genome, and three ORFs have been predicted in SmDV, containing two major gene cassettes. The ORF1/2 encode the SmDV NS proteins, and the Parvo_NS1 conversed domain (Accession number: cl24009) was predicted in ORF2. ORF3 encode the SmDV VP proteins, and Denso_VP4 (Accession number: cl03545) conserved domains was predicted. By RACE and RT-PCR, one and two introns have been confirmed in SmDV NS and VP mRNAs, respectively. The intronic regions also reported in other insect densoviruses, which probably improve the viral protein production by RNA alternative splicing (41). The genome structure of SmDV was shown in **Figure 1B**.

**Figure 2:**
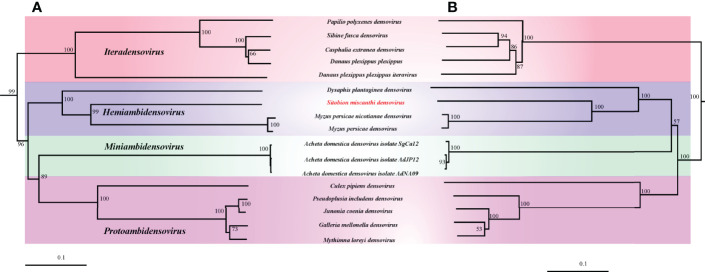
The phylogenetic analysis of *Densovirinae*. **(A)** Phylogenetic tree constructed by NS1 protein sequences. **(B)** Phylogenetic tree constructed by VP protein sequences.

In this study, the phylogenetic trees constructed with NS1 and VP protein sequences were strictly mirrored (**Figure 2**). In both phylogenetic trees, the monophyly of *Densovirinae* was robustly supported. In them, SmDV was clustered with the other three known aphid DVs with high supports and grouped into the *Hemiambidensovirus* genus clade.

### *In Situ* Hybridization of SmDV

**Figure 3:**
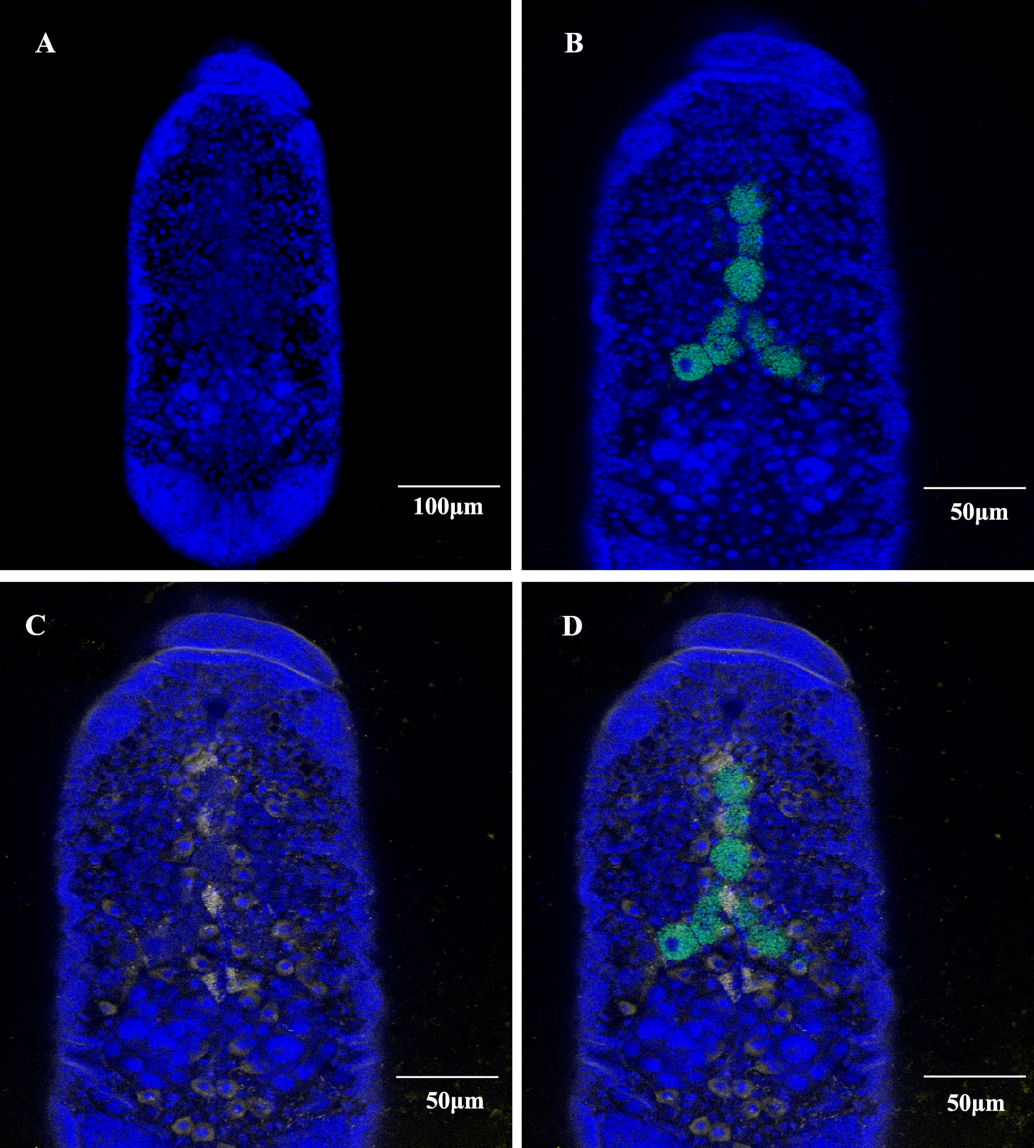
Whole-mount *in situ* hybridization of aphid embryos. *Buchnera aphidicola* (green), SmDV (yellow) and nuclei of aphid cell (blue). **(A)** Color stained aphid cells. **(B)** Distribution of primary bacteriocytes harbouring *B*. *aphidicola*. **(C)** Distribution of cells harbouring SmDV. **(D)** The combined images of **(A–C)**.

The cellular infection pattern of SmDV was observed aphid embryos (**Figure 3**). The nuclei of aphid cells were shown in **Figure 3A**. *B. aphidicola* was observed in the primary bacteriocytes (**Figure 3B**). In aphid, the widely cell distributed pattern of SmDV was revealed in **Figure 3C**. In the **Figure 3D**, we found that SmDV could be harbored in the cells around the bacteriocytes. Control experiments (no-probe and RNase digestion) confirmed the specificity of the observed signals (data not shown).

### Population Dynamics of SmDV With the Development of Aphid

**Figure 4:**
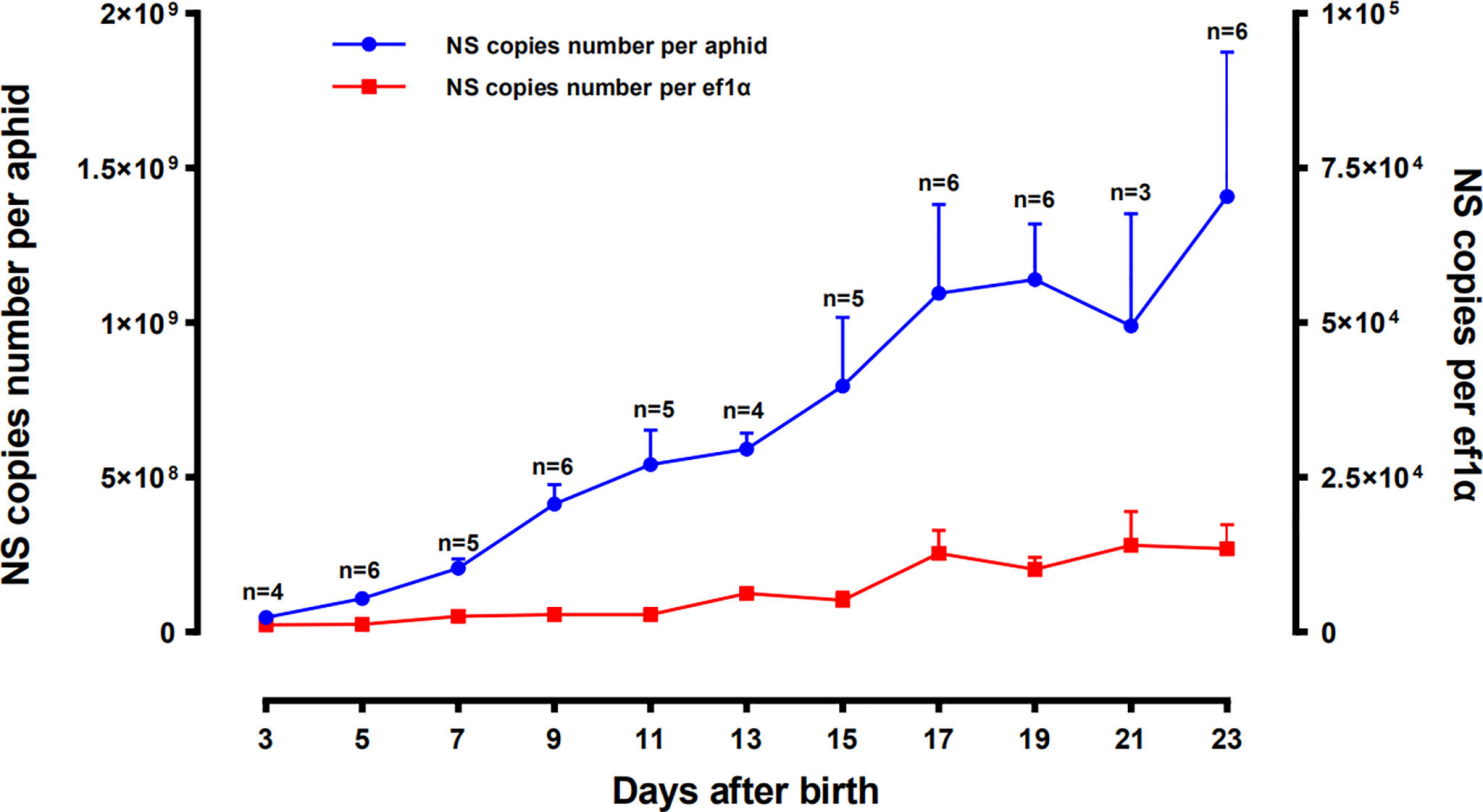
Population dynamics of SmDV along with the development of *Sitobion miscanthi*. Means and positive standard deviations shown; numbers near bars show the replicates.

The concentration changes of SmDV in aphid were uncovered in **Figure 4**. The results indicated that population of SmDV generally increased with the development of aphid, briefly declined and increased again. Aphid 23 day-stage, the last stage we examined here, harbored the highest concentration of SmDV. When normalized by titers of the aphid host gene (*ef1a*), the similar population dynamics of SmDV was observed.

### SmDV Transmissions

In this study, by feeding on the contaminate wheat leaves, SmDV could be horizontally transmitted to the natural negative *S. miscanthi* with about 96% (24/25) transmission rate. Furthermore, the offspring of the above positive SmDV transmitted aphids were collected to evaluate the SmDV vertical transmission rate in the newly SmDV infected aphids. The results indicated that the vertical transmission rate was about 91.7% (22/24) in them. However, we found that the two negative samples were both collected from the same aphid colony. The samples collected from other five aphid colonies were all positive of SmDV. It indicated that the vertical transmission rate in the newly SmDV transmitted aphids were divergent, which probably due to the initial virus concentration they obtained during their feeding. However, the stable infection in the SmDV positive aphid colonies were uncovered in the following detections 100% (60/60).

### The Effects of SmDV Infection on the Aphid Gene Expression

**Figure 5:**
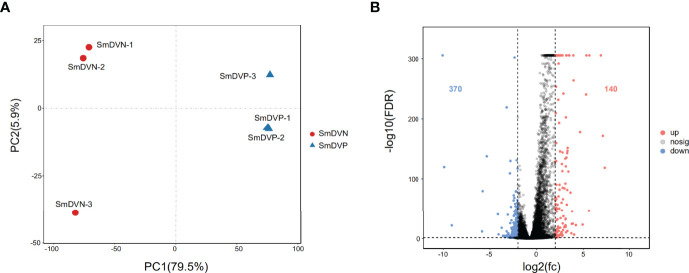
The differentially expressed genes (DEGs) analysis. **(A)** Principal component analysis (PCA) of the samples using the gene expression matrix. **(B)** Volcano plot of differentially expressed genes (DEGs). SmDNV, SmDV negative samples; SmDVP, SmDV positive samples.

**Figure 6:**
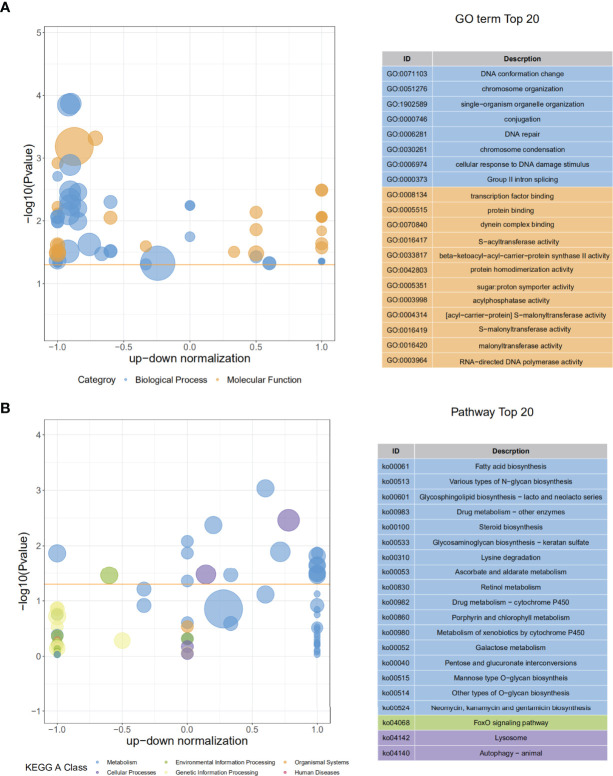
The GO and KEGG enriched results. **(A)** The top 20 of GO enrichment of DEGs. **(B)** The The top 20 of KEGG enrichment of DEGs.

In RNA-seq, the SmDV positive and negative samples were well distinguished in PCA analysis (**Figure 5A**). It indicated that SmDV infection strongly changed the gene expression pattern in aphids. Based on the DEGs filtered threshold, 510 unigenes were selected, with 140 were significantly upregulated and 370 were significantly downregulated in SmDV positive aphids compared to the SmDV negative aphids (**Figure 5B**). Furthermore, the top 20 upregulated and downregulated DEGs were filtered by the fold change of expressions between SmDV positive and negative samples (**Table S4**). The enriched GO terms and KEGG pathways of DEGs were provided in **Figure 6**. The enriched GO terms of the DEGs were all grouped into biological process or molecular function. DNA conformation change, chromosome organization, transcription factor binding, protein binding and dynein complex binding were the top 5 significantly enriched GO terms (FDR < 0.05; **Figure 6A**). In KEGG analysis, Fatty acid biosynthesis, Lysosome, Various types of N-glycan biosynthesis, Glycosphingolipid biosynthesis-lacto and neolacto series, and Drug metabolism-other enzymes were the top 5 significantly enriched the pathways (FDR < 0.05; **Figure 6B**).

**Figure 7:**
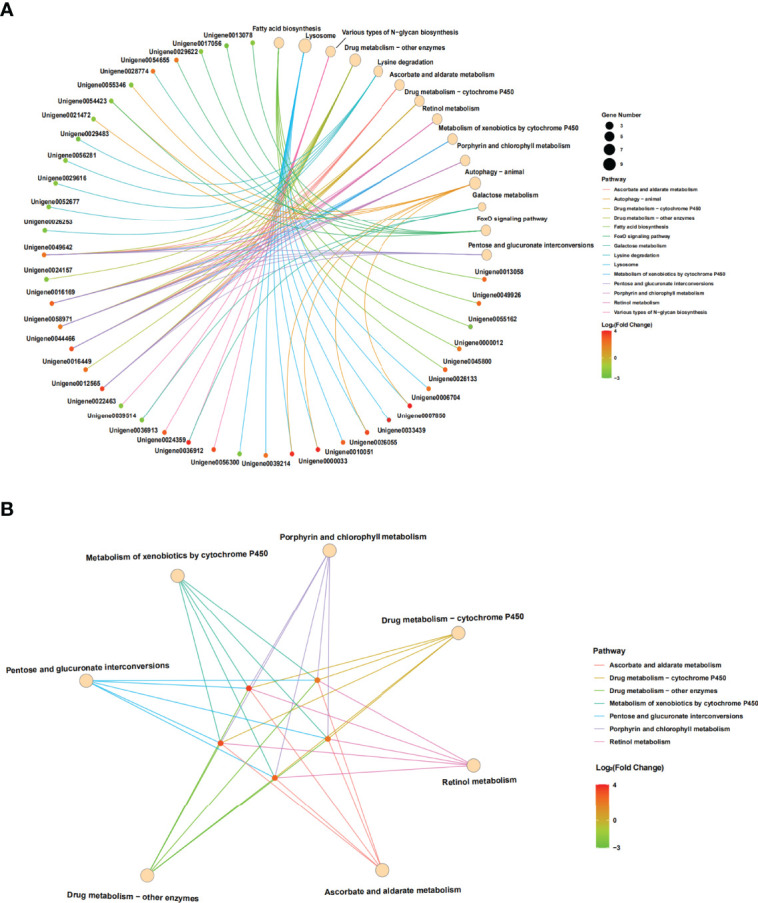
The results of gene-concept network. **(A)** Full map of gene-KEGG pathway network. **(B)** The gene-KEGG pathway mapped with core genes.

**Figure 8:**
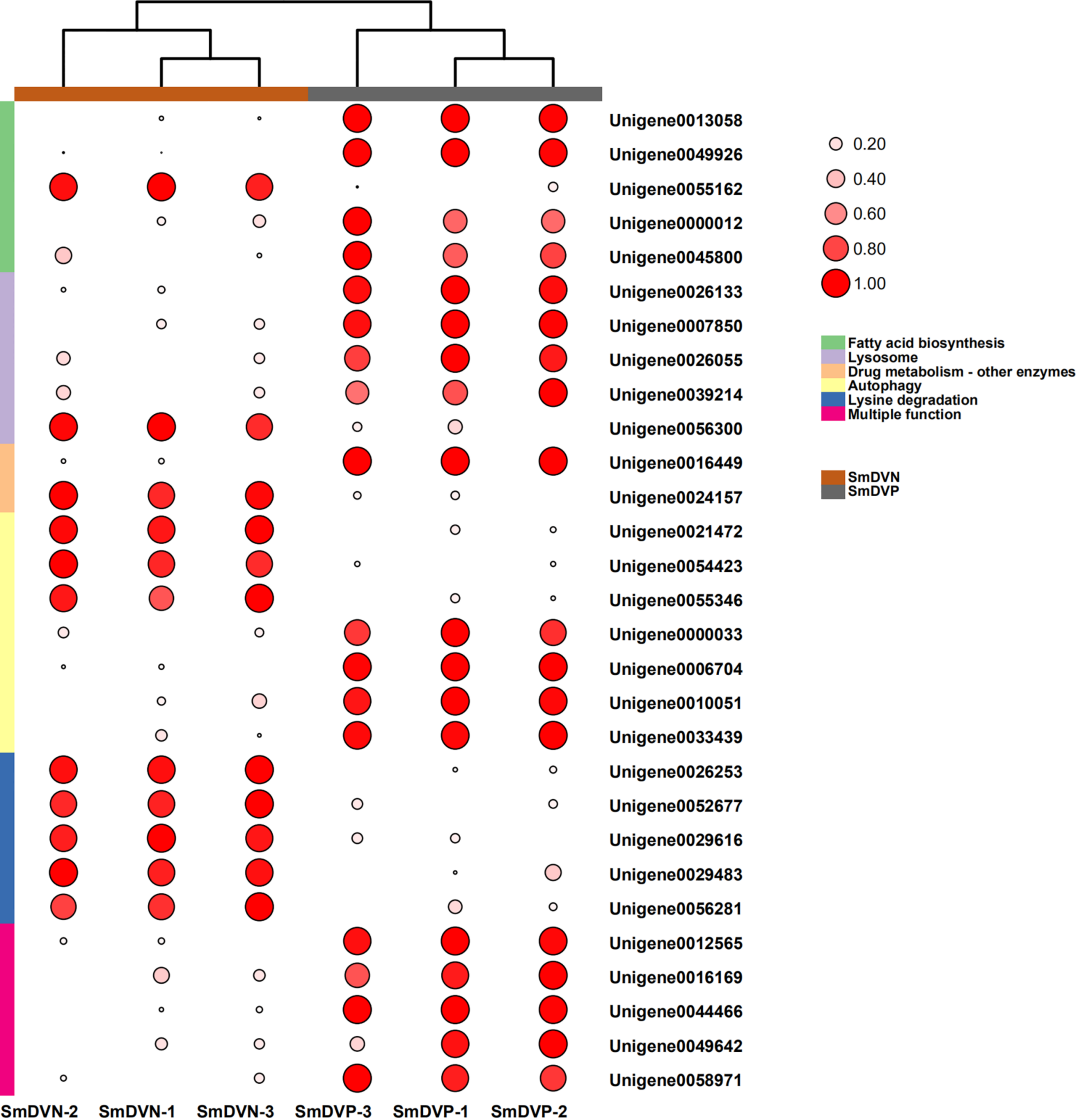
The expression patterns of DEGs related to the significantly enriched pathways. Log2 RPKM values are shown by the color and area of the circles.

Furthermore, the interactions between DEGs and the significantly enriched pathways were visualized in the gene-concept network (**Figure 7A**). Five core DEGs were filtered as they shared in multiple significantly enriched pathways, including Metabolism of xenobiotics by cytochrome P450, porphyrin and chlorophyll metabolism, Drug metabolism-cytochrome P450, Retinol metabolism, Ascorbate and aldarate metabolism, Drug metabolism-other enzymes, and Pentose and glucuronate interconversions (**Figure 7B**). The expression patterns of DEGs in the significantly enriched pathways among the samples were visualized in heat maps (**Figure 8**). The results showed that the five core DEGs (grouped into the multiple function type) all significantly upregulated by SmDV infection. On the contrary, the DEGs enriched in the Lysine degradation pathway were significantly downregulated by SmDV infection. The expression patterns of the DEGs uncovered by RNA-seq were further verified by qRT-PCR (**Figure S2**).

## Discussions

*Hemiambidensovirus* is one the new genera proposed in *Densovirinae*. It consists of two densovirus species identified in aphid, DplDV1and MpDV1. In this study, the monophyly of *Hemiambidensovirus* is strongly supported. SmDV is clustered into the *Hemiambidensovirus* clade, sharing close relationships with DplDV1 and MpDV1. Genomic evidence indicates that SmDV shares the common features of *Hemiambidensovirus*, such as has an ambisense genome, contains two ITRs, and possess a conserved PLA2 domain in its VP protein sequence. Furthermore, the NS1 protein sequence of SmDV share less than 85% identity to the known densoviruses. Hence, SmDV is a new member of *Hemiambidensovirus*.

*In situ* hybridization reveals that, relative to the *B. aphidicola*, SmDV can infect diverse cells, even the ones around primary bacteriocytes. Moreover, the aphid secondary symbionts are also reported housed by the sheath cell and secondary bacteriocytes near the primary bacteriocytes (42). It indicates that the possible interactions would happen between SmDV and aphid bacterial symbionts. Contrary to *B. aphidicola*, the population dynamic of which generally increase in the early aphid development stages, declines in older stages. In aphid, the concentration changes of SmDV shares the similar dynamics with that of aphid secondary symbionts, and highest concentration of them occurs at the older aphid stage (17). As secondary symbionts are common in aphids, the possible antagonistic relationship between them and SmDV should be examined.

In aphids, densovirus can be both vertically and horizontally transmitted (21, 22). In this study, using RT-PCR, SmDV is detect in the RNA samples of the YY-strain aphids feeding wheat leaves (data not shown). It indicates that SmDV keeps its activity in the wheat leaves. Hence, the contaminate wheat leaves would improve the horizontal transmission of SmDV between aphids. Our results show that transmission of SmDV *via* plant feeding is efficient and the stable infected population can be established.

Previous studies indicated that most of the DVs were pathogenic to their natural hosts (43, 44). Hence, DVs are protein agents in the pathogen vectors and pest control (45–47). Recently, mutualistic DV has also been reported in *Helicoverpa armigera* (48), and rapid spread in the wild moth populations (49). In aphids, MpDV1 negatively affects the fitness of *M. persicae* (21), DplDV1 induces winged morphs in *D. plantaginea* (22). In insects, Toll, immunodeficiency (IMD), c-Jun N-terminal kinase (JNK), and Janus kinase/Signal transducers and activators of transcription (JAK/STAT) are the four main pathways in recognition of invasive microbes (50), including viruses. In aphids, due to the long-time evolutionary history with the bacterial symbionts, they lack a number of crucial genes related to immune responses (51). Hence, the underlying mechanisms resistant to microbes in aphids are fascinated. In this study, KEGG enriched results show that the intrinsic antiviral mechanism in insects, such as autophagy pathway is also activated subjected to SmDV (52–54). The results of gene-concept network show that five significantly upregulated DEGs are shared in seven KEGG pathways, which probably be the other potential antiviral pathways in aphids. In these pathways, ascorbate and aldarate metabolism has been reported responding to the plant virus infection in *M. persicae* (55). Moreover, the degradation products of chlorophyll metabolism would serve antiviral function when binding to their receptor proteins (56). A recent study shows that the p450 related drug metabolism pathways can regulate the replication of plant virus in insect (57). In vertebrates, retinoids play key roles in the process of embryonic development and tissue regeneration (58, 59). Retinoid system is also harbored by invertebrates (60). In insects, retinoids are also crucial for their developments (61–63). An early microarray study shows that genes related to retinol metabolism in *Bombyx mori* are stimulated by the infection of cytoplasmic polyhedrosis virus (BmCPV) (64). Recently, by RNA-seq, retinol metabolism pathway has been revealed responding to the infections of Zika virus in mosquito (65), and Nuclear Polyhedrosis Virus in moth (66). It implies that retinol metabolism probably plays a common antiviral role in insects. In human, retinoids would inhibit the replication of measles virus (MeV), by up-regulating elements of innate immune response, i.e., Type 1 interferons (IFN-I) (67). Herein, five core DEGs shared between retinol metabolism and other six KEGG pathways of *S. miscanthi*, are up-regulated by the SmDV infections. Hence, subject to SmDV, it is not clear whether retinol metabolism play a possible antiviral role itself or by an indirect way, as observed in human. In future, the effects of retinoids on the antiviral process in insects are necessary to be investigated.

## Data Availability Statement

The datasets presented in this study can be found in online repositories. The names of the repository/repositories and accession number(s) can be found below: NCBI, accession ID: PRJNA820959 and MF083940.

## Author Contributions

TL and HL collected the samples and designed the experiments. TL and SL analyzed the data. TL, YW, and PX wrote the paper. All authors contributed to the article and approved the submitted version.

## Funding

This work was supported by Major public welfare scientific research project of Henan Province (Grant No. 201300111600), Fund for Distinguished Young Scholars from Henan Academy of Agricultural Sciences (Grant No. 2020JQ05), Henan Province Key R&D and Promotion Project (Grant No. 222102110317) and the National Natural Science Foundation of China (Grant No. 31601897). The funders had no role in study design, data collection and analysis, decision to publish, or preparation of the manuscript.

## Conflict of Interest

The authors declare that the research was conducted in the absence of any commercial or financial relationships that could be construed as a potential conflict of interest.

## Publisher’s Note

All claims expressed in this article are solely those of the authors and do not necessarily represent those of their affiliated organizations, or those of the publisher, the editors and the reviewers. Any product that may be evaluated in this article, or claim that may be made by its manufacturer, is not guaranteed or endorsed by the publisher.

## Supplementary Material

Supplementary Figure 1 The secondary structure of SmDV inverted terminal repeat (ITR).

Supplementary Figure 2 The verification of the DEGs expression by qPCR.
