# Supplementary figures and images for: Identification of a Novel Densovirus in Aphid, and Uncovering the Possible Antiviral Process During Its Infection
Source: Front Immunol. 2022 Jun 9;13:905628. doi: 10.3389/fimmu.2022.905628 (PMC9218065; doi:10.3389/fimmu.2022.905628)

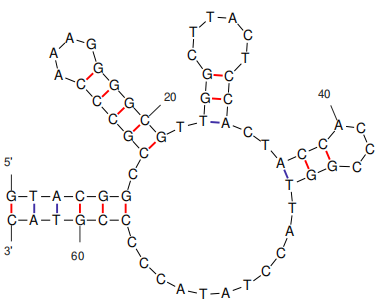

Supplement: Supplementary Figure 1 — The secondary structure of SmDV inverted terminal repeat (ITR). [file Image_1.tif]

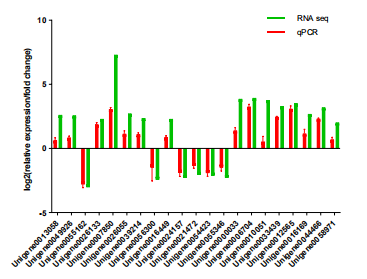

Supplement: Supplementary Figure 2 — The verification of the DEGs expression by qPCR. [file Image_2.tif]
